# Supplementary material for: Decoding and reprogramming fungal iterative nonribosomal peptide synthetases
Source: Nat Commun. 2017 May 23;8:15349. doi: 10.1038/ncomms15349 (PMC5457498; doi:10.1038/ncomms15349)
Supplement: Supplementary Information — Supplementary Figures, Supplementary Tables. [file ncomms15349-s1.pdf]

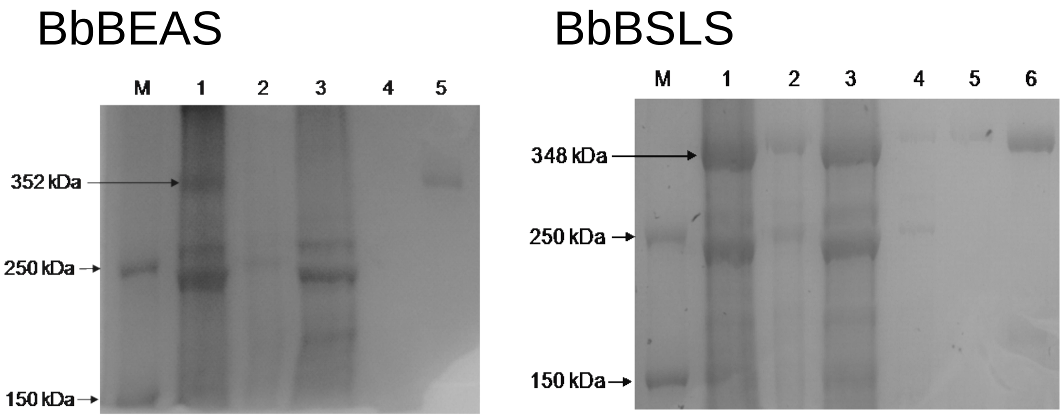

Supplementary Figure 1 | SDS-PAGE analysis of the expression and purification of BbBEAS from *S. cerevisiae* BJ5465-NpgA/pDY37 (left) and BbBSLS from *S. cerevisiae* BJ5465-NpgA/pDY42 (right). M: Protein ladder, 1: Soluble fraction of the lysate, 2: Insoluble fraction of the lysate, 3: Flow through, 4-6: Eluents by buffer A with 10, 100 and 250 mM imidazole, respectively.

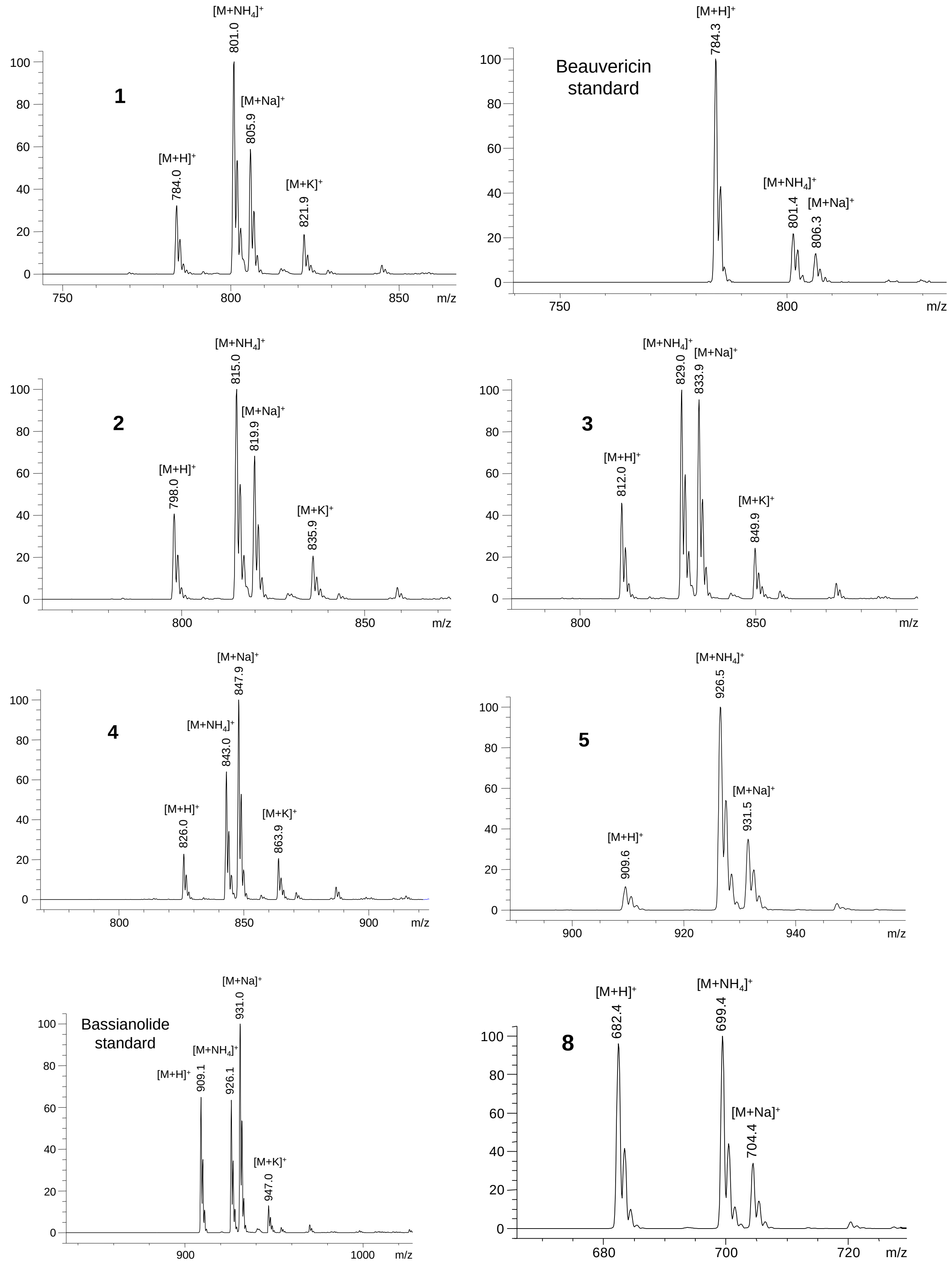
1
[M+H]+ 784.0
[M+NH4]+ 801.0
[M+Na]+ 805.9
[M+K]+ 821.9
m/z
Beauvericin standard
[M+H]+ 784.3
[M+NH4]+ 801.4
[M+Na]+ 806.3
m/z
2
[M+H]+ 798.0
[M+NH4]+ 815.0
[M+Na]+ 819.9
[M+K]+ 835.9
m/z
3
[M+H]+ 812.0
[M+NH4]+ 829.0
[M+Na]+ 833.9
[M+K]+ 849.9
m/z
4
[M+H]+ 826.0
[M+NH4]+ 843.0
[M+Na]+ 847.9
[M+K]+ 863.9
m/z
5
[M+H]+ 909.6
[M+NH4]+ 926.5
[M+Na]+ 931.5
m/z
Bassianolide standard
[M+H]+ 909.1
[M+NH4]+ 926.1
[M+Na]+ 931.0
[M+K]+ 947.0
m/z
8
[M+H]+ 682.4
[M+NH4]+ 699.4
[M+Na]+ 704.4
m/z

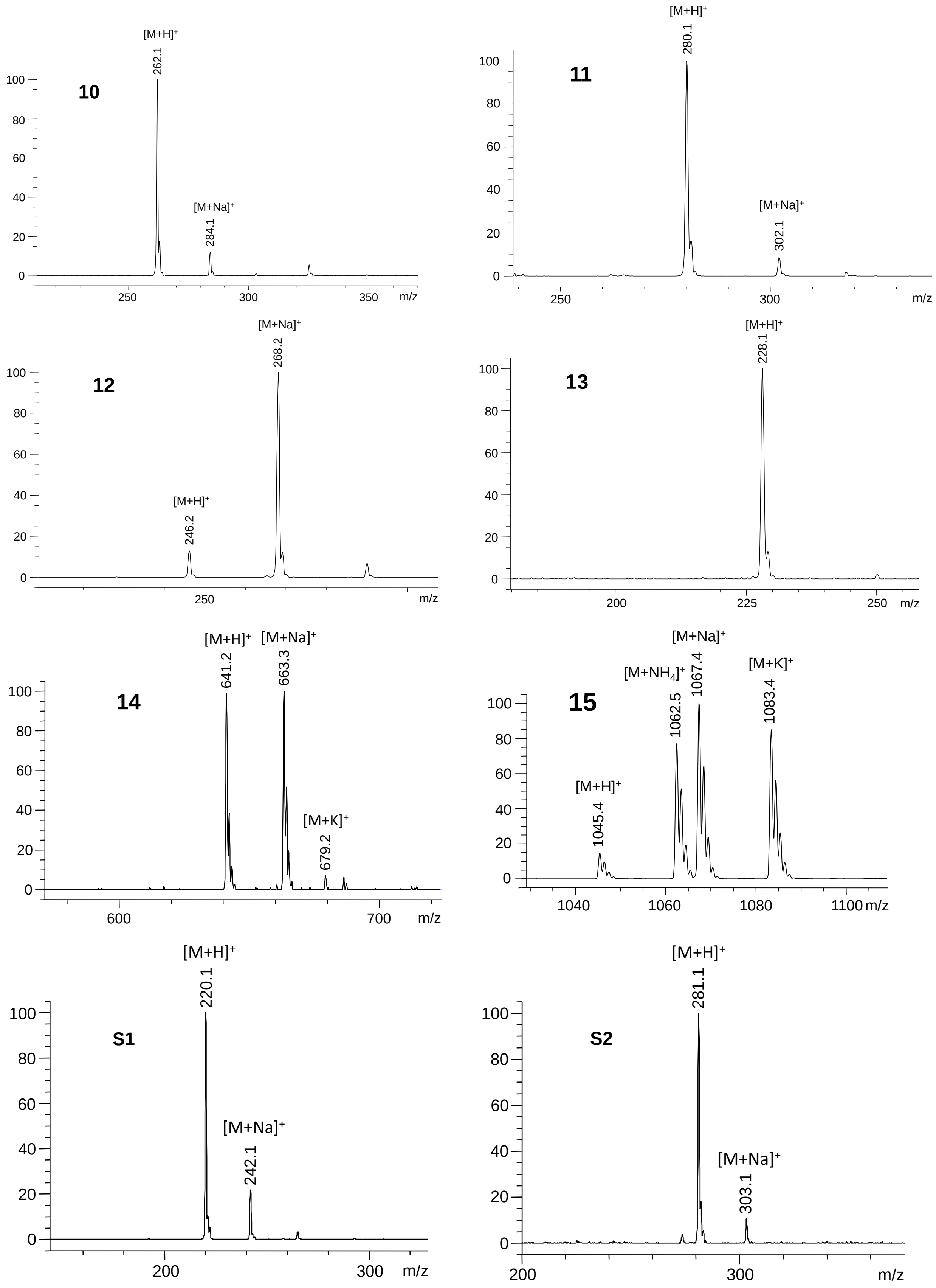
10
[M+H]+
262.1
[M+Na]+
284.1
m/z
11
[M+H]+
280.1
[M+Na]+
302.1
m/z
12
[M+Na]+
268.2
[M+H]+
246.2
m/z
13
[M+H]+
228.1
m/z
14
[M+H]+
641.2
[M+Na]+
663.3
[M+K]+
679.2
m/z
15
[M+Na]+
1067.4
[M+NH4]+
1062.5
[M+K]+
1083.4
[M+H]+
1045.4
m/z
S1
[M+H]+
220.1
[M+Na]+
242.1
m/z
S2
[M+H]+
281.1
[M+Na]+
303.1
m/z

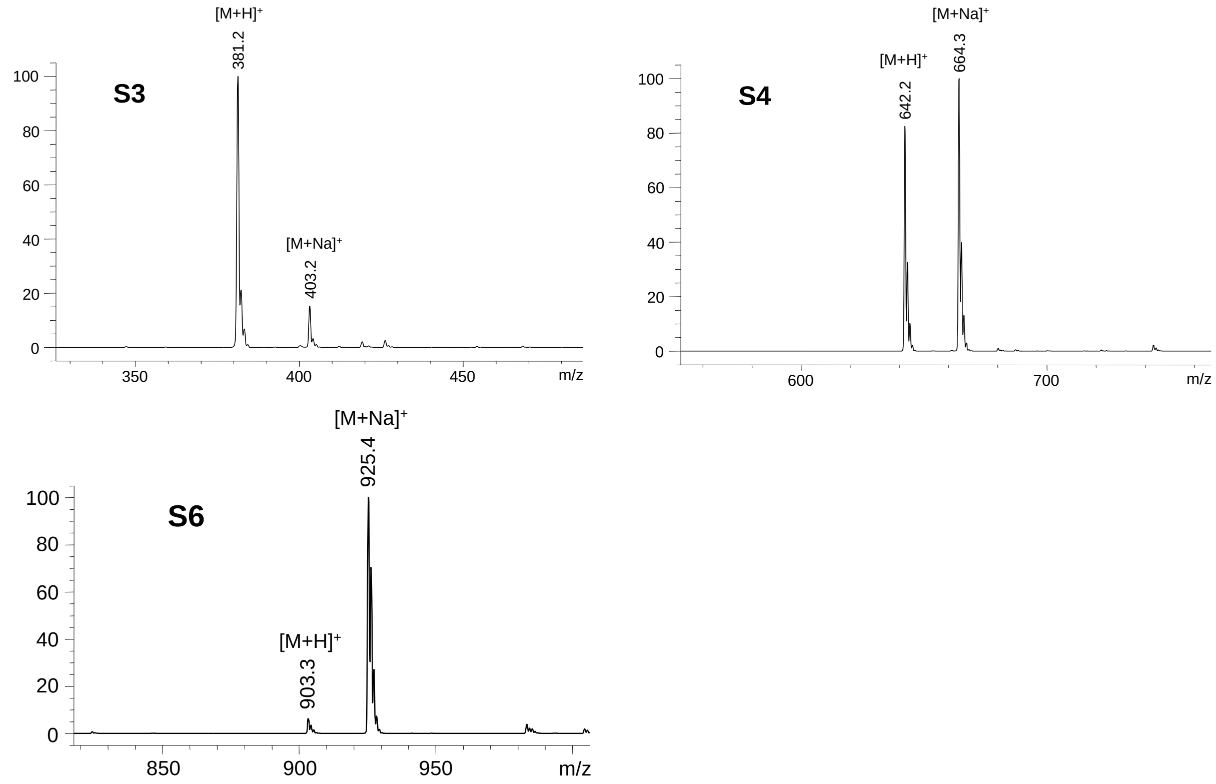

Supplementary Figure 2 | ESI-MS (+) spectra of compounds synthesized (S1 and S2) or biosynthesized (1-5, 8, 10-15, S3, S4 and S6) in this work and the authentic samples of beauvericin and bassianolide.

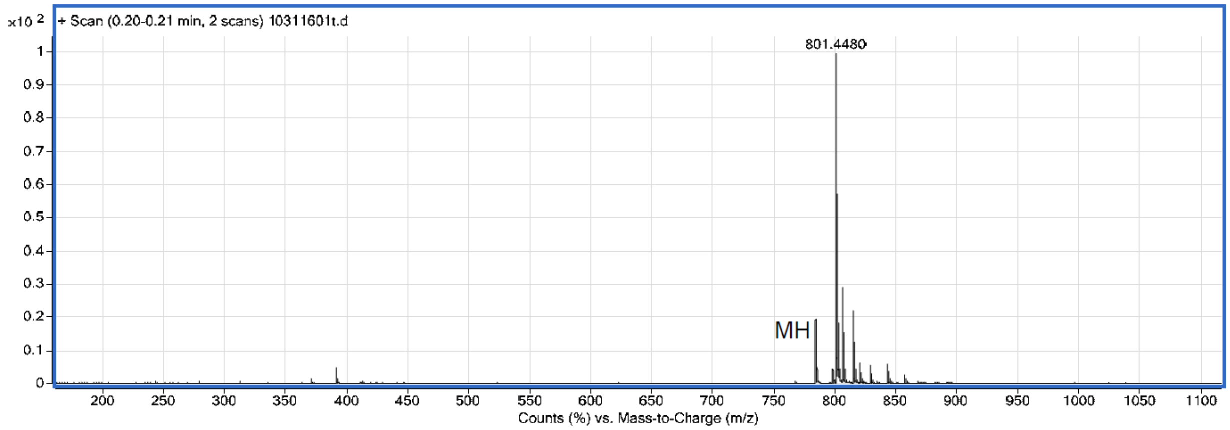

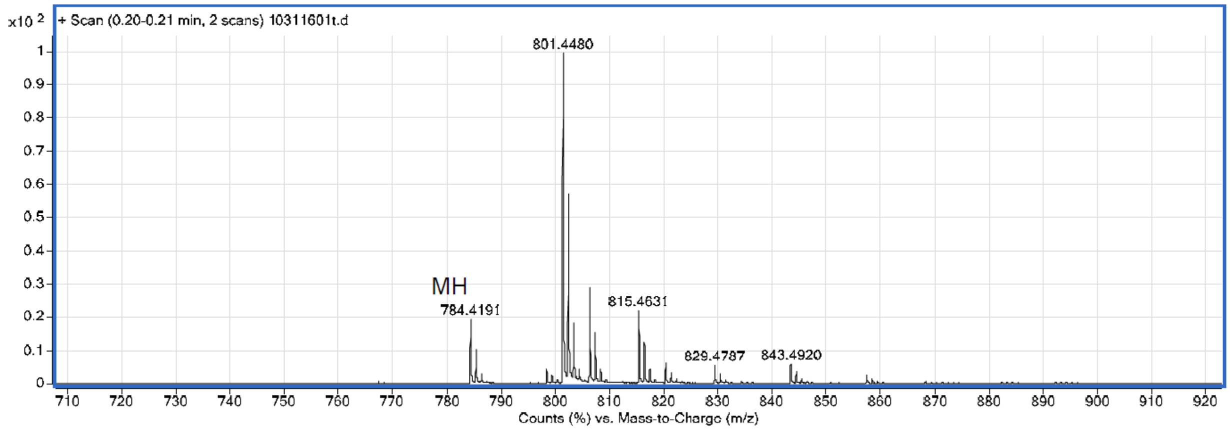

Supplementary Figure 3 | High resolution ESI-MS of 1 generated in the *in vitro* reaction of BbBEAS.

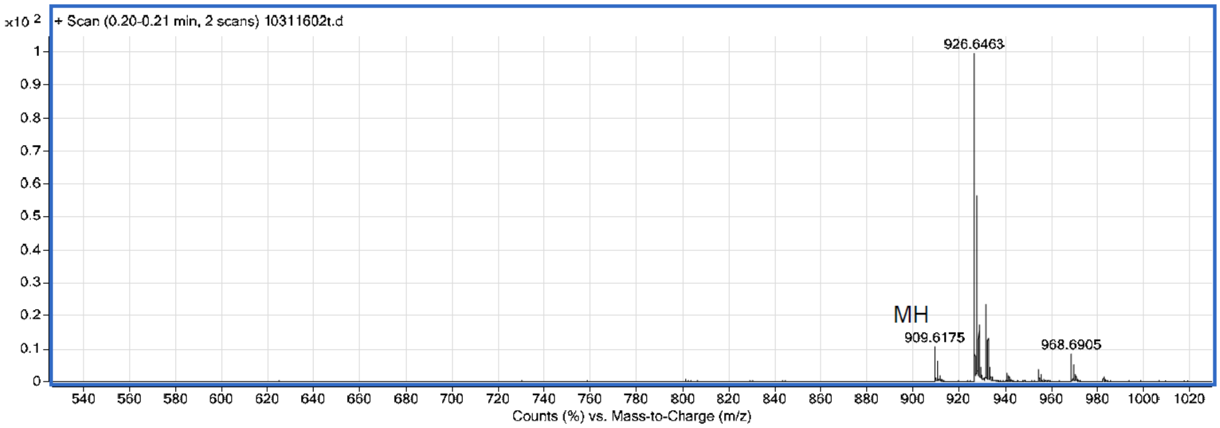

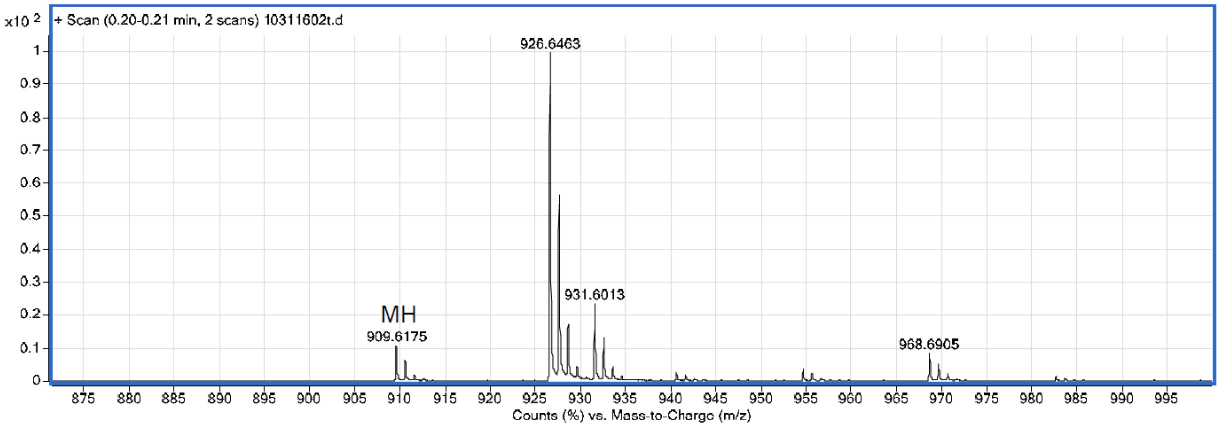

Supplementary Figure 4 | High resolution ESI-MS of 5 generated in the *in vitro* reaction of BbBSLS.

```
BbBEAS-T2a VALCEEATATFGMQ-VGISDHFFKLGGHSLLATKLISRVGDRLKARLTVKDVFDHPIFSELA
BbBSLS-T2a VMLCEELTKTFEMD-VNITDDFFQLGGHSLLATRLVARISHRLGARLTVKDVFDYPVFSELA
BbBEAS-T2b TMLCEEFANVLGMD-VGVTDNFFDLGGHSLMATKLAARIGRRLNTTISVKEVFEHPIVFQLA
BbBSLS-T2b AMLCEEFANILGMD-VGITDNFFDLGGHSLMATRLAARIGHRLNTTISVKDIFSHPVIFQLS
TycC-T3    SKLAEIWERVLGVSGIGILDNFFQIGGHSLKAMAVAAQVHREYQVELPLKVLFAQPTIKALA
GrsB-T3    GKLEEIWKDVLGLQRVGIHDDFFTIGGHSLKAMAVISQVHKECQTEVPLRVLFETPTIQGLA
```

Supplementary Figure 5 | Amino acid sequence alignment of the twin T_2 domains of BbBEAS and BbBSLS with TycC-T_3 and GrsB-T_3. TycC-T_3 is a T domain from TycC (tyrocidine synthetase 3, GenBank accession number AAC45930) and GrsB-T_3 is from GrsB (gramicidin S synthetase 2, GenBank accession number BAA06146). The conserved motif (I/L)GG(D/H)SL is highlighted and the key Ser residue is boxed.

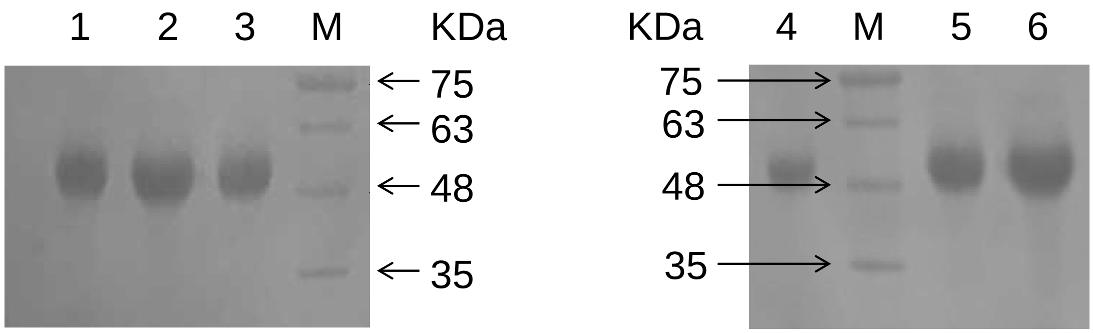

Supplementary Figure 6 | SDS-PAGE analysis of the purified C_1, C_2, C_3 and MT domains from *E. coli* BL21(DE3). M: Protein ladder, 1: $C_{1(BbBEAS)}$, 2: $C_{3(BbBEAS)}$, 3: $C_{3(BbBEAS\text{-}H2901A)}$, 4: $MT_{(BbBEAS)}$, 5: $C_{3(BbBSLS)}$, 6: $C_{2(BbBEAS)}$.

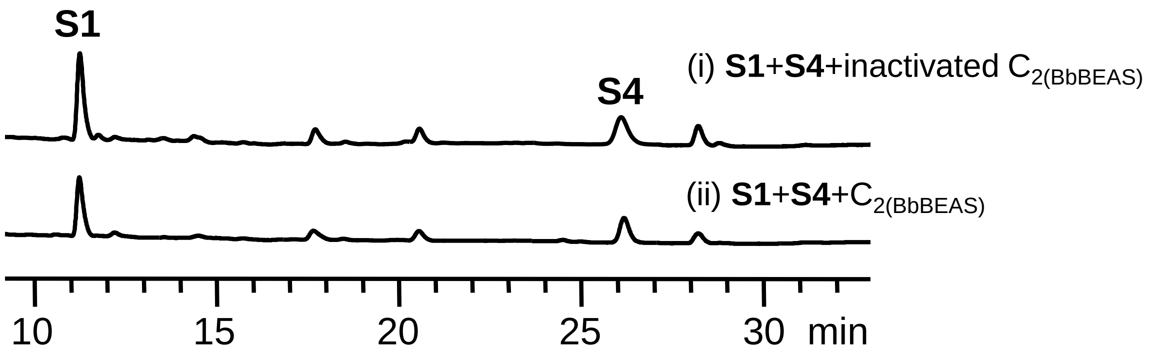

Supplementary Figure 7 | HPLC analysis of the reaction of $C_{2(BbBEAS)}$ with S1 and S4. (i) **S1**+**S4**+inactivated $C_{2(BbBEAS)}$; (ii) **S1**+**S4**+$C_{2(BbBEAS)}$.

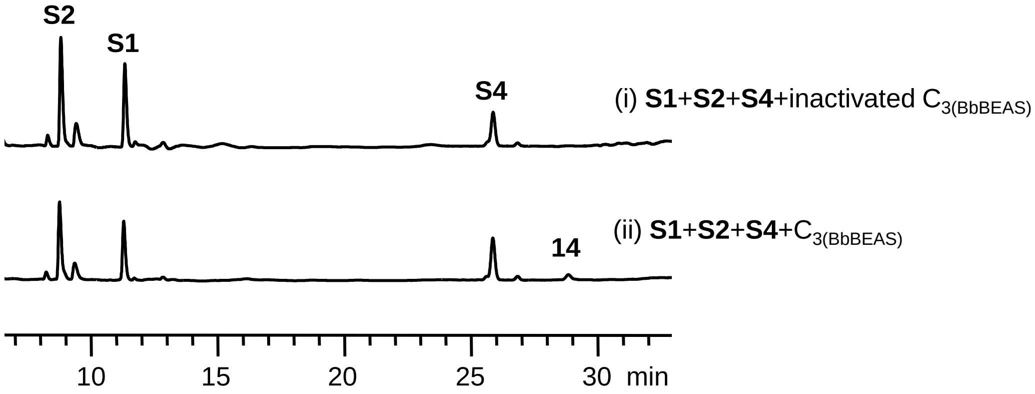

Supplementary Figure 8 | HPLC analysis of the reaction of $C_{3(BbBEAS)}$ with S1, S2 and S4. (i) **S1**+**S2**+**S4**+inactivated $C_{3(BbBEAS)}$; (ii) **S1**+**S2**+**S4**+$C_{3(BbBEAS)}$.

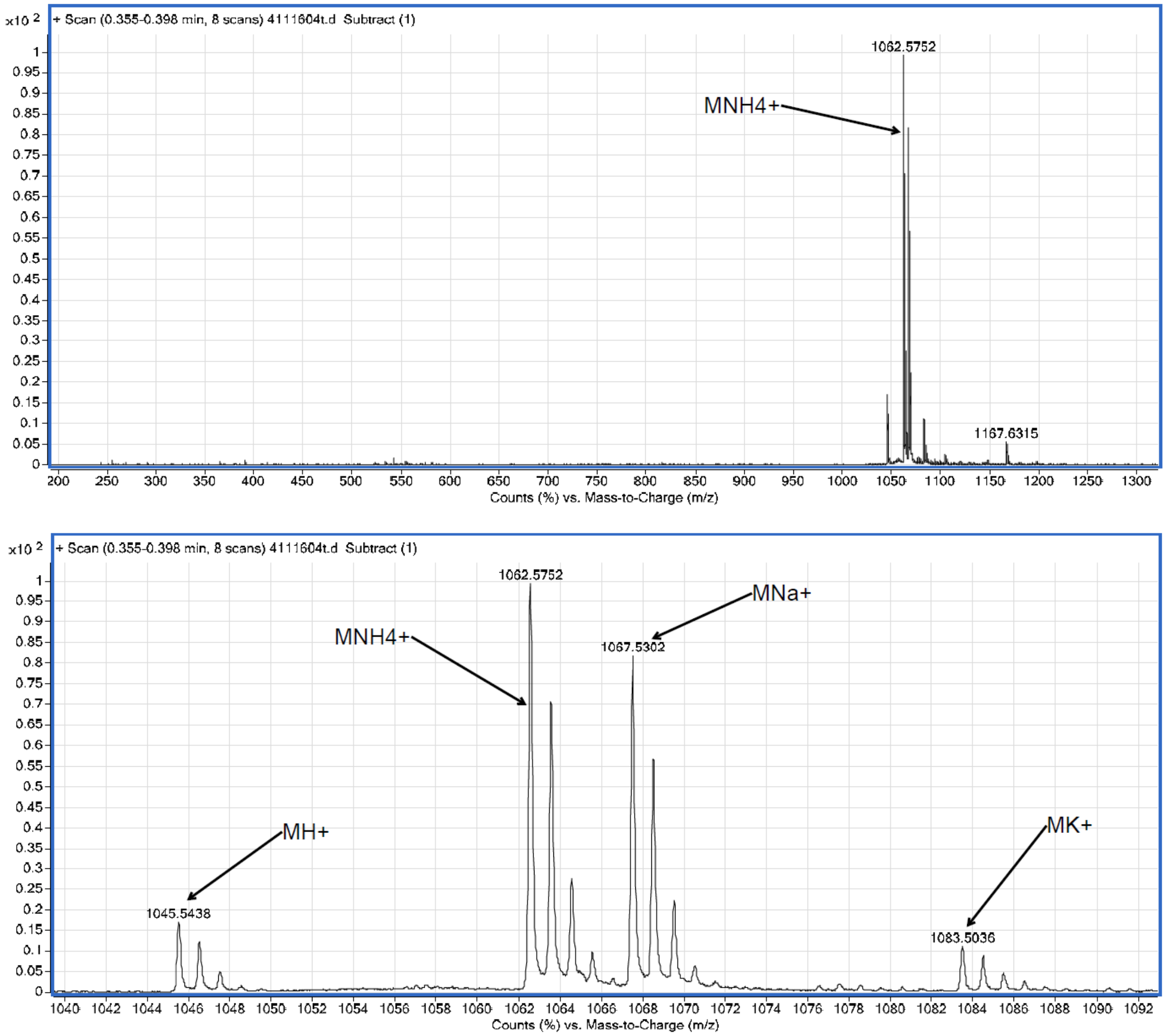

Supplementary Figure 9 | High resolution ESI-MS of FX1 (15).

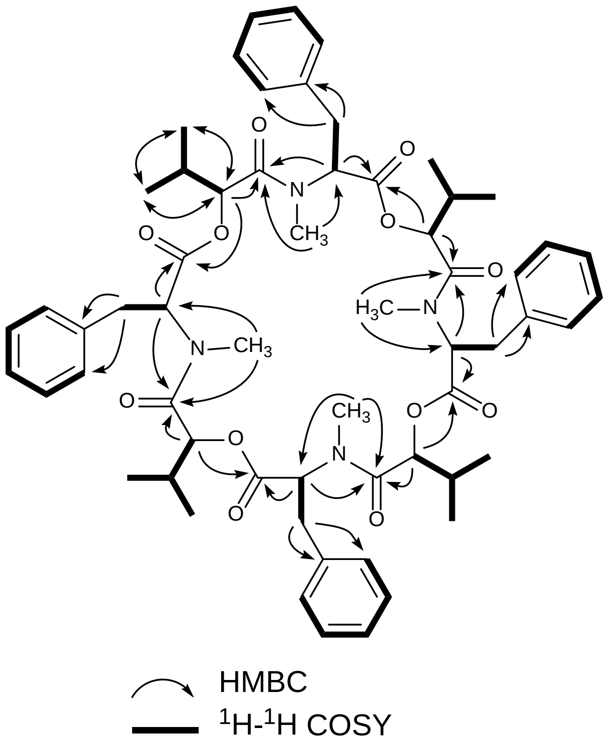

Supplementary Figure 10 | Selected HMBC and ^{1}H-^{1}H COSY correlations for FX1 (15).

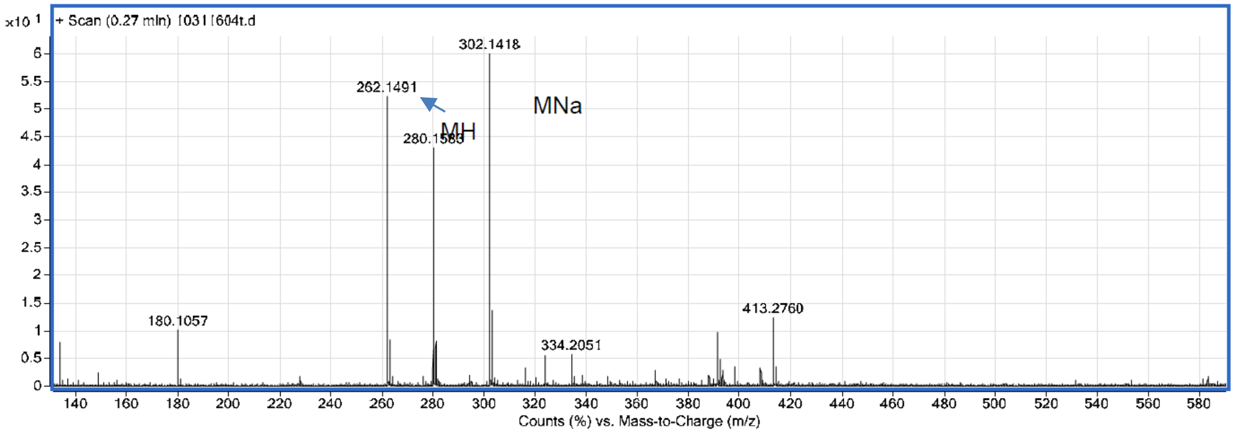

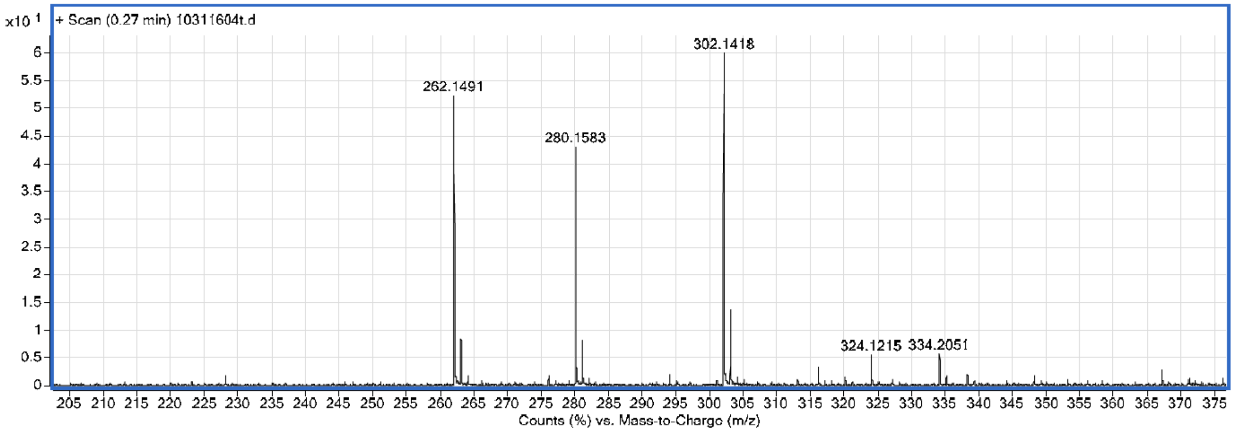

Supplementary Figure 11 | High resolution ESI-MS of D-Hiv-*N*-Me-L-Phe (11) obtained by hydrolysis of 15 with 0.1 N NaOH.

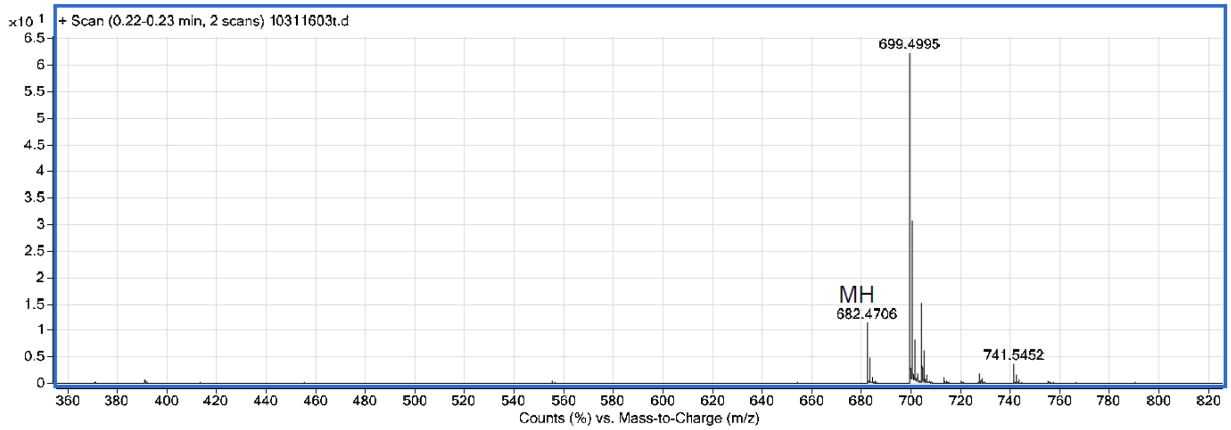

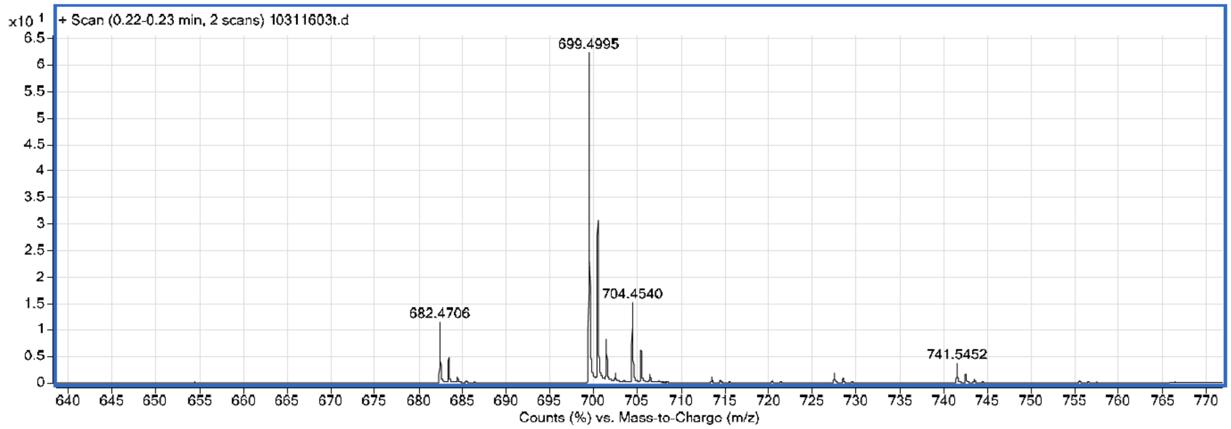

Supplementary Figure 12 | High resolution ESI-MS of enniatin C (8) generated by C_1-A_1-T_1-C_2-A_2-MT-T_{2a}-$T_{2b(BbBSLS)}$-$C_{3(BbBEAS)}$ in the yeast.

Supplementary Table 1 | NMR data for 8, 10, 11 and 15.

Compound	NMR data
Enniatin C (**8**)	^{1}H NMR (300 MHz, $CDCl_3$): δ 5.27 (3H, dd, *J* = 5.4,10.8 Hz), 4.93 (3H, d, *J* = 8.7 Hz), 3.10 (9H, s), 2.22 (3H, m), 1.75 (3H, m), 1.65 (3H, m,) 1.47 (3H, m), 1.00 (9H, d, *J* = 6.6 Hz), 0.94 (9H, d, *J* = 6.3 Hz), 0.93 (9H, d, *J* = 6.9 Hz), 0.91 (9H, d, *J* = 6.6 Hz)
Cyclo-D-Hiv-*N*-Me-L-Phe (**10**)	^{1}H NMR (300 MHz, $CDCl_3$): δ 7.31-7.34 (3H, m), 7.10-7.14 (2H, m), 4.40 (1H, t, *J* = 4.5 Hz), 3.30 (1H, dd, *J* = 4.1, 14.1 Hz), 3.19 (1H, dd, *J* = 4.5, 14.1 Hz), 3.02 (3H, s), 3.00 (1H, d, *J* = 2.4 Hz), 2.28-2.35 (1H, m), 0.84 (3H, d, *J* = 7.2 Hz), 0.76 (3H, d, *J* = 6.6 Hz)
	^{13}C NMR (75 MHz, $CDCl_3$): δ 167.4, 165.6, 134.2, 129.8 (2×), 129.3 (2×), 128.3, 81.4, 62.8, 37.2, 32.5, 29.8, 18.7, 15.2
D-Hiv-*N*-Me-L-Phe (**11**)	^{1}H NMR (300 MHz, $CDCl_3$): δ 7.10-7.34 (5H, m), 4.41 (1H, t, 4.3), 3.28 (1H, dd, *J* = 4.1, 14.0 Hz), 3.21 (1H, dd, *J* = 4.6, 14.0 Hz), 3.02 (3H, s), 3.00 (1H, d, *J* = 2.1 Hz), 2.27-2.37 (1H, m), 0.84 (3H, d, *J* = 7.1 Hz), 0.76 (3H, d, *J* = 6.9 Hz)
	^{13}C NMR (75 MHz, $CDCl_3$): δ 168.1, 165.8, 134.2, 129.9 (2×), 129.4 (2×), 128.7, 81.5, 63.0, 37.3, 32.6, 29.9, 18.8, 15.3
FX1 (**15**)	^{1}H NMR (500 MHz, CD_3OD): δ 7.21-7.39 (20H, m, phenyl-CH of Phe), 6.02 (1H, dd, *J* = 3.5, 13.4 Hz, α-CH of Phe), 5.98 (1H, dd, *J* = 3.0, 12.9 Hz, α-CH of Phe), 5.70 (1H, dd, *J* = 2.9, 12.8 Hz, α-CH of Phe), 5.11-5.42 (4H, m, α-CH of Hiv), 5.20 (1H, dd, *J* = 4.0, 11.7 Hz, α-CH of Phe), 3.54-3.58 (2H, m, β-CH of Phe), 3.44-3.50 (2H, m, β-CH of Phe), 3.22 (3H, s, *N*-CH_3), 3.06 (3H, s, *N*-CH_3), 3.08 (3H, s, *N*-CH_3), 2.90-3.10 (4H, m, β-CH of Phe), 2.81 (3H, s, *N*-CH_3), 1.51-1.70 (4H, m, β-CH of Hiv), 0.89 (3H, d, *J* = 6.1 Hz, γ-CH_3 of Hiv), 0.71 (3H, d, *J* = 6.1 Hz, γ- CH_3 of Hiv), 0.65 (3H, d, *J* = 6.0 Hz, γ-CH_3 of Hiv), 0.60 (3H, d, *J* = 7.3 Hz, γ-CH_3 of Hiv), 0.58 (3H, d, *J* = 7.2 Hz, γ- CH_3 of Hiv), 0.55 (3H, d, *J* = 6.9 Hz, γ-CH_3 of Hiv), 0.48 (3H, d, *J* = 6.6 Hz, γ-CH_3 of Hiv), 0.28 (3H, d, *J* = 6.1 Hz, γ- CH_3 of Hiv)
	^{13}C NMR (125 MHz, CD_3OD): δ 173.4, 172.8, 172.1, 171.9, 171.0, 170.9, 170.7, 170.5 (C=O), 138.6, 138.3, 138.0, 137.5 (γ-C of Phe), 131.0 (2×CH), 130.9 (2×CH), 130.3 (4×CH), 130.1 (2×CH), 130.0 (2×CH), 129.9 (2×CH), 129.7 (2×CH), 128.2 (2×CH), 128.1 (2×CH) (phenyl-CH of Phe), 78.9, 77.2, 76.7, 76.5 (α-CH of Hiv), 62.0, 59.7, 59.4, 58.8 (α-CH of Phe), 35.9, 35.8, 32.6, 32.5 (CH_2 of Phe), 29.1, 29.0, 27.0, 26.9 (β-CH of Hiv), 31.8, 31.7, 31.2, 31.1 (*N*-CH_3 of Phe), 20.4, 19.5, 19.2, 18.9, 18.4, 17.0, 16.5, 15.4 (CH_3 of Hiv)

Supplementary Table 2 | Primers used in this study.

No.	Primer	Sequence	Restriction site(s)
1	$C_{3(BbBEAS)}$-NheI-F	5'-aaGCTAGCatggagctgggtcagttggagag-3'	*Nhe*I
2	BbBEAS-B-PmlI-wostop-R	5'-aaCACGTGcaaagccgagtttagactct-3'	*Pml*I
3	BbBEAS-M1-NdeI-F	5'-aaCATATGgagccgctcaaaaatgt-3'	*Nde*I
4	$T_{2b(BbBEAS)}$-withstop-PmeI-R	5'-aaGTTTAAACtcaaataccgccatgtgcacatt-3'	*Pme*I
5	$C_{1(BbBEAS)}$-withstop-PmeI-R	5'-aaGTTTAAACtcagtcctcgcgagtcacaatgt-3'	*Pme*I
6	BbBSLS-M1-NdeI-F	5'-aaCATATGgagccacccaacaacgc-3'	*Nde*I
7	$C_{1(BbBSLS)}$-withstop-PmeI-R	5'-aaGTTTAAACtcacagtggttcggaattccagc-3'	*Pme*I
8	$T_{2a}T_{2b}C_{3(BbBEAS)}$-NheI-F	5'-aaGCTAGCatgctggctcgtagagcccggac-3'	*Nhe*I
9	$T_{2b}C_{3(BbBEAS)}$-NheI-F	5'-aaGCTAGCatgcgcgaggggctgcaaaacgt-3'	*Nhe*I
10	$C_{3(BbBSLS)}$-NdeI-F	5'-aaCATATGgaggtttctcaattggaaag-3'	*Nde*I
11	$A_2MT_{(BbBEAS)}$-wistop-PmeI-R	5'-aaGTTTAAACtcatgcaacctcaatgtcgctga-3'	*Pme*I
12	$A_{1(BbBEAS)}$-NdeI-SpeI-F	5'-aaCATATGACTAGTctccagattctgcaagagtc-3'	*Nde*I, *Spe*I
13	$T_{2a(BbBEAS)}$withstop-PmeI-R	5'-aaGTTTAAACtcacatcgtttccatttcattgc-3'	*Pme*I
14	$T_{2b(BbBSLS)}$-wostop-PmlI-R	5'-aaCACGTGcatgtcctgtgaaaagttga-3'	*Pml*I
15	BbBSLSM1-SpeI-F	5'-aaACTAGTatggagccacccaacaacgc-3'	*Spe*I
16	BbBSLSM3-PmeI-withstop-R	5'-aaGTTTAAACtcataaagacgcattcaaag-3'	*Pme*I
17	$A_{1(BbBSLS)}$-SpeI-F	5'-aaACTAGTcaccaggttctcgaagagtc-3'	*Spe*I
18	BbBSLS-B-PmlI-wostop-R	5'-aaCACGTGtaaagacgcattcaaagcct-3'	*Pml*I
19	BSLS-M1-5-SpeI	5'-aaACTAGTatggagccacccaacaacgc-3'	*SpeI*
20	BSLS-A2MTwithlinker-withstop-PmlI	5'-aaCACGTGtcagctcagtggaaacgccggcgccgtcggtgg-3'	*PmlI*
21	BSLS-T2aT2bC3linker-5-NdeI	5'-aaCATATGctgtctcgccaggccaaagc-3'	*NdeI*
22	BSLSM3-3-PmeI-withstop	5'-aaGTTTAAACtcataaagacgcattcaaag-3'	*PmeI*
23	BSLS-T2bC3linker-5-NdeI	5'-aaCATATGcgtcaacagttggcctcgaa-3'	*NdeI*
24	BSLS-T2awithlinker-withstop-PmlI	5'-aaCACGTGtcacatggcttccatgtcggtag-3'	*PmlI*
25	BbBEAS-H2901A-F	5'-gagtcttgctttgtctttctgccgccctctat-3'	
26	BbBEAS-H2901A-R	5'-gtactgaagacgcctgcttgagaatggtg-3'	
27	BbBEAS-D179A-F	5'-atcttgcactcgtggccagcaccgttcag-3'	
28	BbBEAS-D179A-R	5'-gaaatacccaaatcagcagttgtttccttg-3'	
29	BbBSLS-H170A-F	5'-ttcttgtatggacattcagcgcctcttttgtcgacagt-3'	
30	BbBSLS-H170A-R	5'-ctcgtctcttggaattgggttctccggtaaggacatat-3'	
31	BbBSLS-H2861A-F	5'-gtccttttgtggctatcagctgctttgtatgatggctt-3'	
32	BbBSLS-H2861A-R	5'-tctcacagacgatgtctgcttcagaaccttgacttgt-3'	
33	BbBEAS-S2591A-F	5'-tcaaactcggcggtcatgctctgcttgctacaaaac-3'	
34	BbBEAS-S2591A-R	5'-aaaagtgatcgctgatgccgacttgcattccaaacgt-3'	
35	BbBSLS-D174A-F	5'-agccactcttttgtcgccagtgcgttcca-3'	
36	BbBSLS-D174A-R	5'-gaatgtccatacaagaactcgtctcttggaa-3'	
37	BbBEAS-S2688A-F	5'-tttttgacctcggtgggcatgcgctcatggcgaca-3'	
38	BbBEAS-S2688A-R	5'-gttgtccgtgactccgacatccatgccaaggacatt-3'	
39	Linker(T_{2a})linker(T_{2b})$T_{2b}C_3$-SOE1-R	5'-cgacgttttgcagcccctcgcgtgcaacctcaatgtcgctg-3'	
40	Linker(T_{2a})linker(T_{2b})$T_{2b}C_3$-SOE2-F	5'-cagcgacattgaggttgcacgcgaggggctgcaaaacgtcg-3'	
41	BbBEAS-AscI7195-F	5'-aaactagatGGCGCGCCctggatatccgccgt-3'	*Asc*I
42	BbBEAS with $T_{2a}T_{2b}C_{3(BbBSLS)}$-F	5'-cccggacgacgacgatggtgcaaaagtcggcacc-3'	
43	BbBEAS with $T_{2a}T_{2b}C_{3(BbBSLS)}$-R	5'-ggtgccgacttttgcaccatcgtcgtcgtccggg-3'	
44	BbBEAS with $T_{2b}C_{3(BbBSLS)}$-F	5'-ctttgaatggtggtggaggccaggacaagaaaga-3'	
45	BbBEAS with $T_{2b}C_{3(BbBSLS)}$-R	5'-tctttcttgtcctggcctccaccaccattcaaag-3'	
46	BbBEAS with $C_{3(BbBSLS)}$-F	5'-acactgcgtttcaactcattcccgccgccgatgc-3'	
47	BbBEAS with $C_{3(BbBSLS)}$-R	5'-gcatcggcggcgggaatgagttgaaacgcagtgt-3'	
48	BbBSLS-BsrGI6843-F	5'-aaTGTACActtgcgcggctcagac-3'	*BsrG*I
49	BbBSLS with $T_{2a}T_{2b}C_{3(BbBEAS)}$-F	5'-ccaaagctatcaaaaagacgaagaaaaagaagcc-3'	

50	BbBSLS with $T_{2a}T_{2b}C_{3(BbBEAS)}$-R	5'-ggcttctttttcttcgtctttttgatagctttgg-3'	
51	BbBSLS with $T_{2b}C_{3(BbBEAS)}$-F	5'-cttcggccggtggtggtcaagcgaagcaagggtc-3'	
52	BbBSLS with $T_{2b}C_{3(BbBEAS)}$-R	5'-gacccttgcttcgcttgaccaccaccggccgaag-3'	
53	BbBSLS with $C_{3(BbBEAS)}$-F	5'-acactgccttccagctcttgtctgttgaagattt-3'	
54	BbBSLS with $C_{3(BbBEAS)}$-R	5'-aaatcttcaacagacaagagctggaaggcagtgt-3'	
55	$C_{1(BbBEAS)}$-withstop-BamHI-R	5'-aaGGATCCtcagtcctcgcgagtcacaatgt-3'	*BamH*I
56	$C_{3(BbBEAS)}$-NheI-pET-F	5'-aaGCTAGCatggagctgggtcagttggagag-3'	*Nhe*I
57	BbBEAS-B-R-BamHI	5'-aaGGATCCtcacaaagccgagtttagactct-3'	*BamH*I
58	$C_{3(BbBSLS)}$-NdeI-pET-F	5'-aaCATATGcagcaatgttttctacgc-3'	*Nde*I
59	BbBSLSM3-BamHI-withstop-R	5'-aaGGATCCtcataaagacgcattcaaag-3'	*BamH*I
60	$C_{2(BbBEAS)}$-NdeI-F	5'-aaCATATGagcggcgattctacgccctc-3'	*Nde*I
61	$C_{2(BbBEAS)}$-HindIII-R	5'-aaAAGCTTtcagctgtcgagctcctttaacc-3'	*Hind*III
62	$MT_{(BbBEAS)}$-NdeI-F	5'-aaCATATGgctgacgatgccgttgagca-3'	*Nde*I
63	$MT_{(BbBEAS)}$-BamHI-R	5'-aaGGATCCtcactgcagccgctgcagcggcc-3'	*BamH*I

Supplementary Table 3 | Plasmids constructed in this study.

Plasmid	Description	Primers
pDY83	$C_{3(bbBeas)}$ in pJET1.2	1, 2
pDY85	*bbBeas*-ΔC_3*(PmeI)* in pJET1.2	3, 4
pDY87	$C_{3(bbBeas)}$ in YEpADH2p-URA3	
pDY88	*bbBeas*-ΔC_3*(PmeI)* in YEpADH2p-TRP1	
pDY92	$C_{1(bbBeas)}$ *in* pJET1.2	3, 5
pDY93	$C_{1(bbBsls)}$ *in* pJET1.2	6, 7
pDY100	$C_{1(bbBeas)}$ *in* YEpADH2p-TRP1	
pDY101	$C_{1(bbBsls)}$ *in* YEpADH2p-TRP1	
pDY104	$T_{2a}T_{2b}C_{3(bbBeas)}$ in pJET1.2	8, 2
pDY105	$T_{2b}C_{3(bbBeas)}$ in pJET1.2	9, 2
pDY106	$C_{3(bbBsls)}$ in pJET1.2	10, 16
pDY108	*bbBeas*-$\Delta T_{2a}T_{2b}C_3$ in pJET1.2	3, 11
pDY109	*bbBeas*-ΔC_1 in pJET1.2	12, 2
pDY111	*bbBeas*-$\Delta T_{2b}C_3$ in pJET1.2	3, 13
pDY112	*bbBsls*-ΔC_3 in pJET1.2	15, 14
pDY113	*bbBsls*-ΔC_1 in pJET1.2	17, 18
pDY114	$T_{2a}T_{2b}C_{3(bbBeas)}$ in YEpADH2p-URA3	
pDY115	$T_{2b}C_{3(bbBeas)}$ in YEpADH2p-URA3	
pDY116	$C_{3(bbBsls)}$ in YEpADH2p-TRP1	
pDY117	*bbBeas*-ΔC_1 in YEpADH2p-URA3	
pDY118	*bbBeas*-$\Delta T_{2b}C_3$ in YEpADH2p-TRP1	
pDY119	*bbBsls*-ΔC_3 in YEpADH2p-URA3	
pDY121	*bbBsls*-ΔC_1 in YEpADH2p-URA3	
pDY122	*bbBeas*-$\Delta T_{2a}T_{2b}C_3$ in YEpADH2p-TRP1	
pDY135	*bbBsls*-$\Delta T_{2a}T_{2b}C_3$ in pJET1.2	19, 20
pDY136	$T_{2a}T_{2b}C_{3(bbBsls)}$ in pJET1.2	21, 22
pDY137	$T_{2b}C_{3(bbBsls)}$ in pJET1.2	22, 23
pDY138	*bbBsls*-$\Delta T_{2b}C_3$ in pJET1.2	19, 24
pDY139	$T_{2b}C_{3(bbBsls)}$ in YEpADH2p-TRP1	
pDY140	$T_{2a}T_{2b}C_{3(bbBsls)}$ in YEpADH2p-TRP1	
pDY141	*bbBsls*-$\Delta T_{2b}C_3$ in YEpADH2p-URA3	
pDY150	*bbBsls*-$\Delta T_{2a}T_{2b}C_3$ in YEpADH2p-URA3	
pDY145	*bbBeas-H2901A* in YEpADH2p-URA3	25, 26
pDY149	*bbBeas-D179A* in YEpADH2p-URA3	27, 28
pDY151	*bbBsls-H170A* in YEpADH2p-URA3	29, 30
pDY152	*bbBsls-H2861A* in YEpADH2p-URA3	31, 32
pDY158	*bbBeas-S2591A* in YEpADH2p-URA3	33, 34
pDY161	*bbBsls-D174A* in YEpADH2p-URA3	35, 36
pDY162	*bbBeas-S2688A* in YEpADH2p-URA3	37, 38
pDY165	*bbBeas*-ΔT_{2a} in pJET1.2	41, 39, 40, 2
pDY173	*bbBeas*-ΔT_{2a} in YEpADH2p-URA3	
pDY183	*bbBeas-S2591A* in pDY162	33, 34

pDY188	*bbBeas with* $T_{2a}T_{2b}C_{3(bbBsls)}$ in pJET1.2	41, 43, 42, 18
pDY189	*bbBeas with* $T_{2b}C_{3(bbBsls)}$ in pJET1.2	41, 45, 44, 18
pDY190	*bbBeas with* $C_{3(bbBsls)}$ in pJET1.2	41, 47, 46, 18
pDY191	*bbBsls with* $T_{2a}T_{2b}C_{3(bbBeas)}$ in pJET1.2	48, 50, 49, 2
pDY192	*bbBsls with* $T_{2b}C_{3(bbBeas)}$ in pJET1.2	48, 52, 51, 2
pDY201	$C_1A_1T_1C_2A_2MT_{(bbBeas)}T_{2a}T_{2b}C_{3(bbBsls)}$ in YEpADH2p-URA3	
pDY203	$C_1A_1T_1C_2A_2MTT_{2a}T_{2b(bbBeas)}$-$C_{3(bbBsls)}$ in YEpADH2p-URA3	
pDY204	$C_1A_1T_1C_2A_2MTT_{2a(bbBeas)}T_{2b}C_{3(bbBsls)}$ in YEpADH2p-URA3	
pDY205	$C_1A_1T_1C_2A_2MTT_{2a(bbBsls)}T_{2b}C_{3(bbBeas)}$ in YEpADH2p-URA3	
pDY215	$C_1A_1T_1C_2A_2MT_{(bbBsls)}T_{2a}T_{2b}C_{3(bbBeas)}$ in YEpADH2p-URA3	
pDY222	*bbBsls with* $C_{3(bbBeas)}$ in pJET1.2	48, 54, 53, 2
pDY224	$C_1A_1T_1C_2A_2MTT_{2a}T_{2b(bbBsls)}$-$C_{3(bbBeas)}$ in YEpADH2p-URA3	
pFC1	$C_{1(bbBeas)}$ in pJET1.2	3, 55
pFC2	$C_{3(bbBeas)}$ in pJET1.2	56, 57
pFC3	$C_{1(bbBeas)}$ in pET28a	
pFC4	$C_{3(bbBeas)}$ in pET28a	
pFC9	$C_{3(bbBsls)}$ in pJET1.2	58, 59
pFC11	$C_{3\ (bbBsls)}$ in pET28a	
pFC44	$C_{3(bbBeas\text{-}H2901A)}$ in pJET1.2	56, 57
pFC46	$C_{3(bbBeas\text{-}H2901A)}$ in pET28a	
pFC62	$C_{2(bbBeas)}$ in pJET1.2	60, 61
pFC63	$C_{2(bbBeas)}$ in pET28a	
pZJ134	$MT_{(bbBeas)}$ in pJET1.2	62, 63
pJCZ21	$MT_{(bbBeas)}$ in pET28a	
